# Can anxiety and race interact to influence face-recognition accuracy? A systematic literature review

**DOI:** 10.1371/journal.pone.0254477

**Published:** 2021-08-06

**Authors:** Isabeau K. Tindall, Guy J. Curtis, Vance Locke

**Affiliations:** 1 School of Psychological Science, University of Western Australia, Perth, Western Australia, Australia; 2 Centre for Transformative Work Design, Curtin University, Perth, Western Australia, Australia; 3 Discipline of Psychology, Murdoch University, Perth, Western Australia, Australia; Victoria University of Wellington, NEW ZEALAND

## Abstract

Wrongful convictions continue to occur through eyewitness misidentification. Recognising what factors, or interaction between factors, affect face-recognition is therefore imperative. Extensive research indicates that face-recognition accuracy is impacted by anxiety and by race. Limited research, however, has examined how these factors interact to potentially exacerbate face-recognition deficits. Brigham (2008) suggests that anxiety exacerbates other-race face-recognition deficits. Conversely, Attentional Control Theory predicts that anxiety exacerbates deficits for all faces. This systematic review examined existing studies investigating the possible interaction between anxiety and face-race to compare these theories. Recent studies included in this review found that both anxiety and race influence face-recognition accuracy but found no interaction. Potential moderators existing in reviewed studies, however, might have influenced their results. Separately, in some studies reviewed, anxiety induced during retrieval impacted recognition, contrasting with the conclusions of previous reviews. Recommendations for future research are given to address moderators potentially impacting results observed previously.

## Introduction

The ability to accurately recognise a face is a vital skill needed for daily functioning. Faces are often the key feature we pay attention to when looking at someone and the primary information source for discerning whether someone we see is known or unknown [1]. Facial recognition ability can be dissociated from both general intelligence and other types of recognition memory, such as memory for objects [2]. Human beings are considered experts at recognising faces; however, this ability is fallible [3, 4]. Because accurately recognising faces is important, extensive research has investigated what affects this ability [3].

Two factors extensively researched regarding their influence on face-recognition accuracy are anxiety [5] and race [6]. The potential interaction between these variables has direct relevance to many real-world situations, such as eyewitness identification [5, 6]. When witnessing a crime, an individual will likely become anxious. Further, the individual committing the crime may be from a different racial group than the witness. This systematic literature review combines the available literature focusing on the influence of anxiety and race on face-recognition accuracy.

### Anxiety

Anxiety can be broadly defined as the anticipation of future threat or danger, characterised by anticipatory cognitive, behavioural, and affective changes in response to the possibility of uncertain future negative events [7]. According to Attentional Control Theory (ACT) [8], anxiety can lead to a reduction in performance on cognitive tasks, such as those used to examine face-recognition accuracy [5, 9]. Specifically, the drop in cognitive performance that occurs within the context of face-recognition occurs via insufficient inhibition of task-irrelevant stimuli while experiencing anxiety [8].

ACT argues that individuals when experiencing anxiety emphasise the processing of threat-related stimuli, either internal (i.e. task-irrelevant thoughts) or external (i.e. processing task-irrelevant stimuli), instead of monitoring performance on the task at hand [8]. Therefore, anxiety impairs attentional control through interfering with the balance between the stimulus-driven system (attends to salient and visible stimuli) and goal-driven systems (monitors task performance), such that the stimulus-driven system is given precedence [10]. The amount of disruption caused by this threat processing is directly proportional to the amount of anxiety experienced, such that the higher the level of anxiety, the larger the impairment in attentional control, and subsequently, the greater the drop in cognitive performance [8]. Different sources of anxiety, such as situational stress, and/or having a strong innate predisposition to becoming anxious increases the tendency to give precedence to processing of task-irrelevant threat stimuli [8].

### Moderators impacting effect anxiety on face-recognition accuracy

Within the literature, several moderators potentially influencing how anxiety impacts performance have been identified. Anxiety is not a unidimensional concept, but rather, is considered to be multidimensional [11, 12]. Anxiety is commonly separated into the dimensions of state anxiety; the transient anxiety response, and trait anxiety; the stable tendency to become state anxious [12]. The State-Trait Anxiety Inventory (STAI) is often used to measure these forms of anxiety [13]. However, recently, it has been argued that anxiety should be further separated into the dimensions of cognitive anxiety; anxiety symptoms related to thought processes, and somatic anxiety; anxiety related to physiological symptoms [11]. Recent studies have suggested that these dimensions differently impact performance on cognitive tasks [14–16]. For instance, anxiety inductions that induce anxiety through physiological anxiety symptoms, such as the 7.5% CO_2_ Challenge, due to inducing an unconditioned anxiety response [17, 18], have been argued to disrupt face-recognition accuracy to a larger magnitude than cognitive anxiety inductions. Therefore, the type of anxiety induction used may moderate the strength of the anxiety response, and therefore, the effect of anxiety on face-recognition accuracy [19, 20].

Another moderator that has been found to impact the effect of anxiety on face-recognition accuracy is face-stimuli valence [21–25]. Individuals when experiencing heightened state and/or trait anxiety give preferential processing to negative (i.e. angry, fear or sad) face-stimuli as compared to non-negative stimuli (i.e. happy or neutral) [26, 27]. Greater processing therefore increases recognition accuracy for this type of stimuli when experiencing anxiety [9]. For instance, in numerous studies, high trait anxious individuals [26, 28], and those experiencing heightened state anxiety [26], had increased face-recognition accuracy for negative stimuli. Therefore, it is important to either control for stimuli-valence through using face-stimuli of the same valence, or purposely measuring the impact of stimuli-valence on face-recognition accuracy.

### Memory phase of induced anxiety

In addition to seeing anxiety as a multidimensional concept, and measuring the impact of face-stimuli valence, it is also important to consider the phase of memory formation in which the anxiety was induced, as this can also influence face-recognition accuracy through disrupting recognition [19, 29, 30]. Recognition consists of two stages: encoding, where information is stored into memory [31], and retrieval, where previously-encoded information is accessed [32]. According to Deffenbacher et al. [5], anxiety may only impact face-recognition accuracy when induced during encoding. Applying the principles of ACT [8], anxiety can interfere with encoding through the preferential allotment of cognitive resources to worrying thoughts; with this impairing task performance [5, 10, 33]. Theoretically, anxiety experienced during retrieval might also inhibit memory [19] through interfering with previously-encoded memories [34]. However, although possible in theory, evidence for the impact of anxiety on retrieval in terms of face-recognition accuracy has been inconsistent [19, 20, 29, 35].

### Race

Race also affects face-recognition accuracy [6]. Termed the Own-Race Bias (ORB) in the literature, the ORB is the tendency to have lower face-recognition accuracy, specifically, fewer hits (correctly identifying a previously seen face) and hit-rate (probability of correctly responding affirmatively to seeing a face previously) for other-race faces. Individuals also have increased false alarms (incorrectly perceiving a face as previously seen) and false-alarm rate (probability of incorrectly indicating seeing a face previously) for individuals of another race [6, 36, 37]. Further, individuals have a reduced d-prime (*d′*: accuracy of correctly identifying a target as previously seen or unseen) and more liberal response criterion (response bias: tendency to respond liberally or strictly to whether a face has been seen previously, irrespective of accuracy) for other-race faces as compared to own-race faces [6]. The ORB is highly robust, with this effect exhibited in White [6, 36] and Asian [38] individuals viewing White, Asian, Black and Middle Eastern faces [36].

Many models have been created to explain the ORB, broadly categorised into perceptual learning and social cognitive theories (i.e. feature-selection model, see [39]; cognitive disregard model, see [40], ingroup/outgroup model, see [37]; multidimensional face-space model, see [41, 42]). Of these models, research has found most support for the ingroup/outgroup model of face processing (IOM) [37] and the multidimensional face-space model (MDS) [41, 42].

According to the IOM [37], when individuals encounter someone from another race, they automatically categorise them as an outgroup exemplar and therefore impersonalise them when stored into memory [37, 43]. The MDS model suggests that memory for faces is stored in a multidimensional space in which each individual’s identity is represented by a unique point [41, 42]. Perceptual experience with different faces refines the face space so that it can be used to discriminate identities of highly familiar categories, such as human faces [44]. Individuals have more perceptual experience with individuals of their own-race, and so their multidimensional space consists of more own-race, and less other-race examples. This experience leads to better discrimination accuracy for own-race, and increased discrimination errors, for other-race individuals [41].

### Moderators impacting effect of race on face-recognition accuracy

According to the IOM [37], categorisation as part of the outgroup is not specific to race, and ingroup/outgroup classifications according to gender (own-gender bias; see [45]) have also been found [46]. The review article conducted by Herlitz et al. [45] suggests that an own-gender bias is reliability exhibited in research examining face-recognition accuracy. This own-gender bias, however, is only present in female participants, as males do not express this bias [45]. Moderating the impact of perceptual experience on face-recognition accuracy according to the MDS model [41, 42], is the quality of other-race contact [47, 48]. According to the review article by Brigham [49], self-reported interracial contact is positively associated with face-recognition accuracy for other-race faces. Further, the motivation to gain individuating experience with other-race individuals [47] also moderates the influence of perceptual expertise on face-recognition accuracy. Several studies have supported this perspective, as individuals with increased individuating experience or contact, were better able to holistically process other-race faces, and subsequently, had higher other-race face-recognition accuracy [47, 48, 50]. Therefore, own-group biases and the amount of perceptual expertise with other-race indiviudals needs to be considered when examining the impact of race on face-recognition accuracy.

### Anxiety and ORB interaction

As outlined above, anxiety and race can influence face-recognition accuracy when acting separately. Therefore, it is plausible that if these factors were to cooccur, they could interact to alter face-recognition accuracy. Brigham [49] previously suggested that intergroup anxiety and race could interact to exacerbate face-recognition deficits for other-race faces. According to Brigham [49] this interaction occurs through intergroup anxiety reducing face-recognition accuracy for other-race individuals via an increased reliance on categorisation. Anxiety increases categorisation by reducing the cognitive resources available for individuation of faces [51]. Increased reliance on categorisation subsequently increases the reliance on stereotypes for outgroup individuals, as a way of inferring information about the individuals’ racial group [36, 37, 43, 49, 52, 53]. This theory can be adapted to situations involving other types of anxiety, such as anxiety coming from an external source (i.e. state anxiety; [29]) to potentially explain exacerbated other-race face-recognition deficits while highly state anxious. Specifically, Bodenhausen [54] indicated that incidental affect, such as anxiety, can influence outgroup perceptions through increased stereotyping, despite coming from a situation unrelated to the intergroup context.

Unlike the theory adapted from Brigham [49], ACT [8], does not predict an interaction between anxiety and race. Therefore, studies seeking to examine the impact of anxiety on face-recognition accuracy, as per ACT [8], do not normally consider the influence of race on observed results. For instance, a substantial number of previous studies measuring the impact of anxiety on face-recognition accuracy have used mixed-race participants and/or mixed-race stimuli but have failed to examine the influence of race [55–57]. Further, some studies have failed to even record the race of face-stimuli and/or participants altogether (for instance see: [20, 35, 58, 59]).

Although an interaction between anxiety and race has been proposed by Brigham [49], limited research has investigated whether anxiety and race can interact to alter face-recognition accuracy. Because of this, it is important to systematically examine studies that have measured both the impact of anxiety and race on face-recognition accuracy within the same study. Despite the theory by Brigham [49] exisiting within the literature for over a decade, the interaction of these factors has received minimal attention. Further, as outlined above, it is well established that there are several potential moderators of the impact of anxiety and race on face-recognition accuracy. Therefore, when examining studies looking at the interaction between anxiety and race, it is also important to examine the impact of these potential moderators.

### The current study

The purpose of the present systematic literature review, in light of the above, was to examine the possible interaction between anxiety and race in the context of face-recognition accuracy. Because moderators unique to anxiety and race may influence the relationship between these two variables, potential moderators were examined separately. The goals of the current study were explored through three sequential steps. Firstly, this review examined how potential moderators that have been previously identified could influence the effect of anxiety on face-recognition accuracy. For example, how stimuli-valence influences face-recognition accuracy [22]. The influence of anxiety, when induced during either encoding or retrieval, was also examined at this point, as according to ACT [8], anxiety when induced during either encoding or retrieval, can produce memory deficits. The second step examined the influence of moderators specific to race. Potential moderators examined were the influence of possible own-gender biases, as outlined in the IOM [37] and the impact of perceptual expertise and contact on face-recognition accuracy. Lastly, this review directly examined the interaction between anxiety and race, and whether this interaction was in the direction predicted by Brigham [49], or if results supported ACT [8]. We also discuss at this point, how potential moderators identified by the first two steps reduce confidence in results observed.

## Method

### Protocol

This review was conducted following the Preferred Reporting Items for Systematic Literature Review and Meta-Analysis (PRISMA) guidelines for systematic reviews and meta-analyses [60] (see S1 Table) and was preregistered on the Open Science Framework (https://osf.io/ja28t). It must be noted that after peer-review of this article, an additional inclusion criterion was added, such that we have only included studies that examined the interaction between anxiety and race.

### Search procedure

The following electronic databases were searched from their inception through to 7 February 2020: PubMed, PsycINFO, PsycARTICLES, ProQuest, Scopus, Web of Science and Wiley Library (see S1 File for more detail). Search strategies for each database are shown in S1 Appendix. The search was constructed around the differing types of “anxiety” and “face recognition” and were adapted for each database. Due to overlap with eyewitness memory research, searches also included terms such as "stress", "arousal" and "eyewitness memory". Studies measuring related concepts such as stress, however, were only included if they defined this concept such that it would meet the definition of anxiety provided earlier in this paper.

Race was not explicitly searched for to broaden results identified due to extensive variation in ethnic identifications and terms associated with race. Therefore, studies examining how anxiety affects face recognition were examined first, due to this being the first step, and then these studies were further examined according to those examining race, and the interaction between anxiety and race.

Search strategies for databases were cleared by a librarian at the University of Western Australia. Each database search was modified according to available Boolean terms and ability for truncation or proximity searching. Due to common variations in phrasing, terms like “face recognition”, were searched via proximity searching. Where proximity searching was not available or appropriate, the most common phrase variation was searched instead.

Only articles that were in English, full-text, and peer reviewed were included. Abstracts and titles of identified records were screened, and the full articles of potentially eligible studies retrieved. Firstly, all duplicates were removed. Two reviewers then screened the titles and abstracts of the remaining papers individually and obtained full-text of potentially eligible studies. The studies were then checked in detail, with eligible papers included in the systematic literature review. Once included papers were selected, backwards and forward searching of these papers’ references were conducted to find further eligible studies. These articles were then assessed for eligibility according to inclusion and exclusion criteria.

### Inclusion and exclusion criteria

Included studies 1) used an experimental study design, 2) measured face-recognition accuracy as one of the main focuses, 3) face stimuli portrayed any emotional valence (e.g. happy, sad, neutral) from any race (e.g. White, Asian, Black), 4) participants were ≥18 years, with or without a psychiatric diagnosis and from any gender or race, 5) with anxiety explicitly measured, where 6) anxiety was either non-induced (i.e. compared high anxiety to low anxiety), induced through an induction eliciting anxiety, or reduced through a reduction procedure decreasing anxiety and 7) examined the interaction between anxiety and race.

Excluded studies 1) had a main focus on facial expression, object recognition or reaction times, 2) the main focus on anxiety reduction was through a clinical therapy (such as cognitive behaviour therapy), 3) examined artificial facial recognition (i.e., through use of artificial intelligence), and 4) focused on the influence of another clinical diagnosis (such as developmental, intellectual disabilities or traumatic brain injury), in addition to how anxiety affects face recognition.

**Fig 1:**
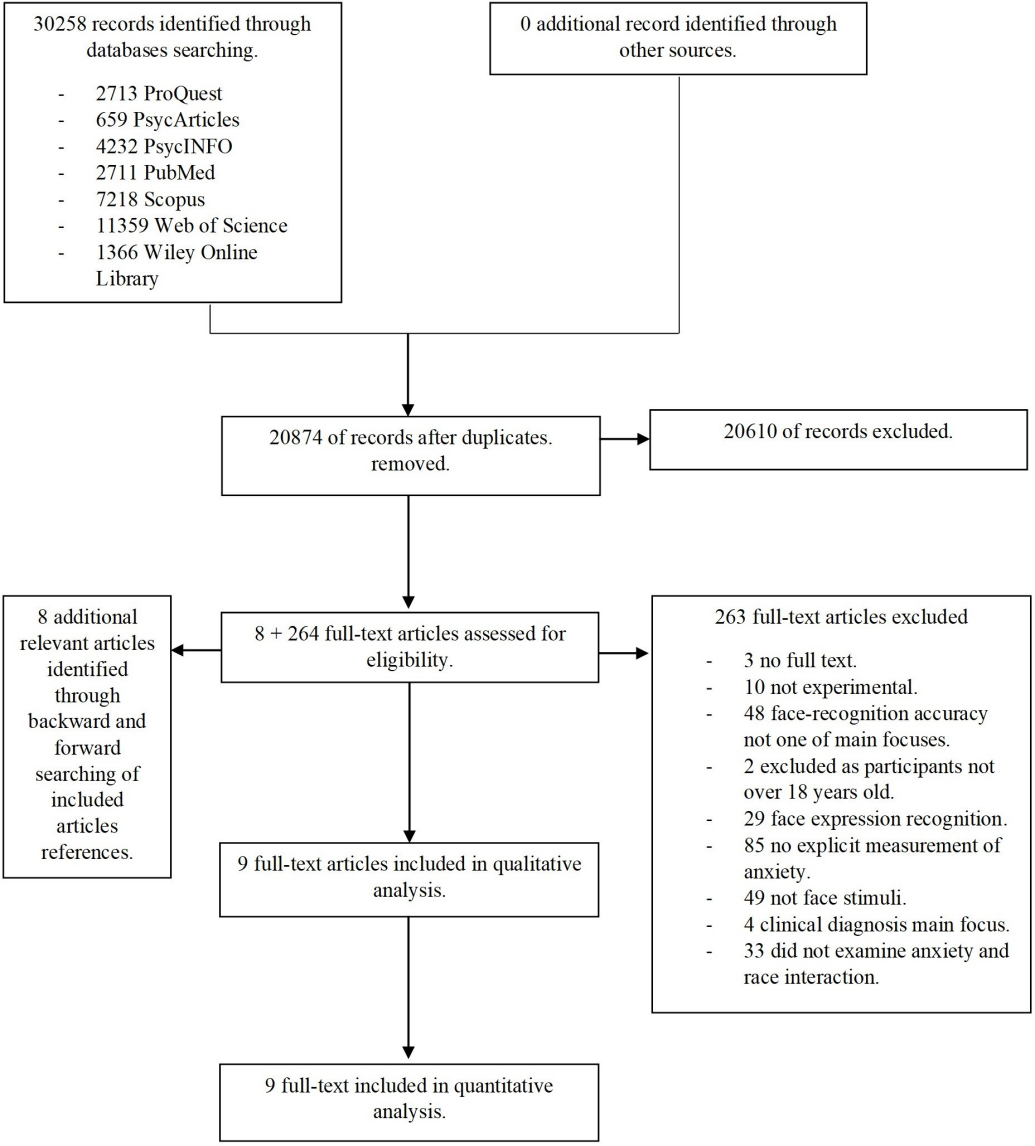
Flowchart of the results for the literature search.

The specific number of included and excluded papers at each step, according to the inclusion and exclusion criteria are listed in Fig 1.

### Data extraction

In line with the first step, data items were extracted according to moderators affecting the influence of anxiety on face-recognition accuracy within studies examining the interaction between anxiety and race. Identified moderators included: the influence of participant gender and stimuli on results (see Table 2). The interaction between stimuli-valence and anxiety, within both clinical and non-clinical populations (see Table 3). An indication of whether anxiety was measured through the STAI, and the influence of inducing anxiety during either encoding or retrieval (also see Table 2). According to the second step, the influence of an own-group bias, specifically gender, was examined. Where applicable, gender was also presented in Table 3 due to overlapping themes. As specified by the third step, the interaction between anxiety and race was examined (see Table 4). Due to limited studies commenting on perceptual expertise and contact (*n* = two), this information was presented in Table 4, as per step two.

Results specific to each of the three steps were recorded according to the signal detection measures they most closely represented [37, 61]; hits/hit-rate, misses, false alarms/false-alarm rate, correct rejections, response bias, and *d′*. Not all studies examined recognition accuracy via signal detection measures or used a closely related measure. These results were therefore labelled ‘general recognition accuracy’ as a way of highlighting that they did not align with signal-detection measures.

#### Risk of bias and quality assessment

An adapted version of the Cochrane Collaboration’s tool for assessing risk of bias [62] assessed bias over five categories (see Table 1, for average scores, and S2 Table for detailed scoring). Selection bias was separated into a) random sequence generation; specifying allocation into different conditions must be described, and b) allocation concealment; stating concealment of allocation into experimental and control conditions should occur before conducting the experiment. Performance bias consisted of a) participants and personnel blinding, which was modified to blinding of participants only, as the experimenter needed to know condition assignment to carry out the anxiety induction/reduction. 3) Detection bias consisting of blinding of outcome assessment; where knowledge of allocation to control or experimental conditions was low risk if this knowledge would not affect the outcome measure. 4) Attrition bias consisting of incomplete outcome data, and 5) reporting bias (selective reporting), defined as reporting bias due to selectively reporting outcome variables. Each study was given a total score, with a maximum score of 6, reflecting the five categories (plus the two sub-categories of category one).

**Table 1:** Characteristics of included studies examining the impact of the potential interaction between anxiety and race on face-recognition accuracy.

Authors	Experiment Design	*N*	Mean age (in years)	% female	Participant Race	Population	Induction Conditions	Anxiety manipulation	Relevant measures	Quality assessment score
**Brigham et al. (1983)** [63]	R-FRT	40	-	50.00	White	University students	Moderate and high arousal (anxiety) condition	Threat of shock	Self-report of anxiety (0–100), HR, finger pulse volume, Racial contact questionnaire and Multifactor Racial Attitude Inventory [64–66]	6
**Johnson et al. (2005)** [30]	R-FRT	89	-	55.06	White	University students	Fear (induced anxiety), control and joy condition	Videos (comedy in joy condition, horror in fear condition and neutral in control conditions)	Retrospective emotion report (including anxiety, joy and fear)	4
**Walker et al. (2008)** [47]	IAT, R-FRT	39	-	53.85	54% White	University students	N/A		25 question survey (questions of relevance on: intergroup anxiety, social contact, and individuating experience)	3
**Horry et al. (2009)** [36]	Exp 2: DPT, R-FRT	127	22.35	65.00	White	University students and University staff	Primed (anxiety) and control condition	Stereotype priming	STAI-T, STAI-S	4
**Wilson et al. (2010)** [67]	R-FRT	Exp 1: 108	-	-	White	University students	Immigration threat (anxiety) and control condition	Immigration threat induction	Emotion report, with questions of relevance including anxiety, worry and threat	4
		Exp 2: 191								
**Wang et al. (2012)** [68]	R-FRT	30	20.17	60.00	East Asian	University students	N/A		Cheek and Buss Shyness Scale [69]	3
**Curtis et al. (2015)** [29]	R-FRT	Exp 1: 51	Exp 1: 22.32	Exp 1: 62.74	White	University students	Exp 1: Anxiety and control condition	Exp 1: Public speech	STAI-T, STAI-S	5
		Exp 2: 110	Exp 2: 21.49	Exp 2: 73.64			Exp 2: Anxiety and control condition	Exp 2: Anagram task		
**Attwood et al. (2015)** [19]	R-FRT	30	-	50.00	White	University staff, University students and Community members	Anxiety and control condition	7.5% CO_2_ Challenge	STAI-T, STAI-S, HR, SBP, DBP	5
**Kikutani (2018)** [38]	R-FRT	87	19.56	71.26	East Asian	University students	N/A		SISST	4

*Note*. Heart rate: HR, Systolic blood pressure: SBP, Diastolic blood pressure: DBP, Not applicable: N/A, Not reported: -, Implicit Association Test: IAT [70], Racial-face recognition task: R-FRT, Dot-probe task: DPT, Social Interaction Self-Statement Test: SISST [71], State and Trait Anxiety Inventory; STAI, STAI-state: STAI-S, STAI-trait: STAI-T [13, 72].

## Results

### Characteristics of included studies

Initially, 30258 records were extracted (see Fig 1). After removing duplicates, reading titles and abstracts, and screening full-texts and searching references, nine articles remained that examined the interaction between anxiety and race. Demographic information related to the included papers, specifically, article authors, experiment design, participant characteristics, induction conditions, anxiety manipulation, and relevant questionnaires given, are presented in Table 1.

As seen in Table 1, all participants either came exclusively from a university sample of students and/or staff (*n* = eight), or from a university sample as well as those from the wider community (*n* = one).

All studies used a racial face-recognition paradigm, where own-race and other-race face-stimuli was used. Two studies, in addition to using a racial face-recognition paradigm, presented face-stimuli to participants using an implicit association test [47] or a dot-probe task [36].

In studies using an anxiety manipulation (six out of nine), the manipulation used across studies was non-uniform. Manipulations used were threat of electric shock [63] (*n* = one), emotive videos [30] (*n* = one), stereotype priming [36] (*n* = one), immigration threat induction [67] (*n* = one), public speaking task [29] (*n* = one), anagram task [29] (*n* = one), and use of 7.5% CO_2_ Challenge [19] (*n* = one). It must be noted that the public speaking task and anagram task were used during experiment one and experiment two of the same study [29]. Most studies using an anxiety manipulation compared the anxiety condition to a control condition [19, 29, 36, 67]. Further, in some studies included, anxiety manipulations, in addition to inducing anxiety symptoms, also induced other affective symptoms, such as fear and increased perception of threat (three out of nine).

Anxiety symptoms across studies were measured using various anxiety scales. Anxiety was measured using the STAI in several studies [19, 29, 36], while other studies used varying rating scales measuring affect (including anxiety symptoms) on a continuum [30, 63, 67]. Three studies measured non-induced anxiety symptoms, with one study [47] measuring intergroup anxiety, one study [38] measuring social anxiety and another [68], shyness (which is associated with social anxiety).

### Risk of bias

As seen in Table 1, most studies were not affected by substantial bias; with an average rating of 4.22 for studies examining the interaction between anxiety and race.

### Potential moderators of anxiety and race

#### Gender

The impact of gender, and interaction of gender with other variables, within included studies are presented in Table 2. All studies recorded participant gender (*n* = nine), face-stimuli gender (*n* = nine) and a majority used a mixed-gender sample of both participants and face stimuli (*n* = five). No studies used the same gender participants and stimuli. Most studies examined the impact of gender on face-recognition accuracy (five out of nine). Further, of studies reporting on gender a minority found an impact of gender on face-recognition accuracy (two out of five). In one of these studies [63], an increased *d′* for female as compared to male face-stimuli occurred in female participants, indicating an own-gender bias. Further, in this study [63], an increased *d′* for female participants also occurred within the moderate-anxiety condition compared to high-anxiety condition. Additionally, in another study [30], individuals had increased recognition accuracy for female own-race stimuli as compared to all other types of face-stimuli.

**Table 2:** Gender of participant and stimuli for included studies, with associated results, including interaction with other variables.

First Author (year)	Mixed Gender Stimuli	Mixed Gender Participants	Results
**Brigham (1983)**	Yes	Yes	***d′*** marginally significantly larger *d′* for female stimuli as compared to male stimuli in female participants; larger *d′* in moderate-anxiety condition than high-anxiety condition in female participants
**Johnson (2005)**	Exp 1 Yes	Yes	**Exp 1 & 2**
	Exp 2 Yes	Yes	**General recognition accuracy** higher recognition accuracy for own-race female stimuli as compared to other-race female stimuli, own-race male stimuli & other-race male stimuli
**Walker (2008)**	Yes	Yes	-
**Horry (2009)**	Exp 2 Male only	Yes	-
**Wilson (2010)**	Exp 1 Male only	Yes	**Exp 1 & 2**
	Exp 2 Male only	Yes	***d′*** no difference between female participants and male participants
**Wang (2012)**	Yes	Yes	-
**Curtis (2015)**	Exp 1 Male only	Yes	**Exp 1 & 2**
	Exp 2 Male only	Yes	**All recognition measures** no difference between female participants and male participants
**Attwood (2015)**	Male only	Yes	-
**Kikutani (2018)**	Yes	Yes	**All recognition measures** no difference between female participants and male participants

*Note*. Not reported: -.

#### Face-stimuli valence

Impact of face-stimuli valence on results are presented in Table 3. Most studies either did not record face-stimuli valence (*n* = four) or used stimuli displaying only a neutral facial expression (*n* = four). Only one study [38] used stimuli of more than one valence and recorded the impact of emotional expression on face-recognition accuracy. Further, this study [38] also recorded the impact of stimuli-valence on the interaction between anxiety and race. All studies used a non-clinical sample of participants. Further, all studies reporting the outcome of the manipulation inducing anxiety were successful. Experiment two of the study by Wilson et al.[67] did not report the effectiveness of the anxiety manipulation, as did the study by Wang et al [68]. Only studies with a reported successful anxiety manipulation are discussed due to the inability of determining anxiety impact without a successful anxiety manipulation.

**Table 3:** Influence of face-stimuli valence and anxiety on face-recognition accuracy, and stage of memory anxiety induced (encoding or retrieval), for included studies.

								Results	
First Author (year)	Measured face valence	Stimuli Valence	Anxiety Diagnosis	Successful anxiety manipulation	Induction/Reduction Type	Memory phase of induction	Used STAI	Impact of stimuli valence	Impact of anxiety
**Brigham (1983)**	No	-	Non-clinical	Yes (female participants only)	Physiological	Encoding	No	-	***d′*** larger *d′* in moderate anxiety condition than high-anxiety condition; marginally significant negative correlation between heart rate and *d′*
									**Hits** increased hits in moderate-anxiety condition than high-anxiety condition
									**False-alarms** More false-alarms in high-anxiety condition than moderate-anxiety condition
**Johnson (2005)**	Exp 1 No	Neutral	Non-clinical	Yes	Psychological	Encoding	No	-	-
	Exp 2 No	Neutral	Non-clinical	Yes	Psychological	Retrieval	No	-	-
**Walker (2008)**	No	Neutral	Non-clinical	Yes	N/A		N/A	-	-
**Horry (2009)**	Exp 2 No	-	Non-clinical	Yes	Psychological	Encoding	Yes	-	***d′*** negative correlation between anxiety and *d′* within control condition as larger *d′* in participants with lower anxiety compared to higher anxiety; positive relationship found within primed (anxiety) condition as larger *d′* in participants with higher anxiety compared to lower anxiety
**Wilson (2010)**	Exp 1 No	Neutral	Non-clinical	Yes (significant increase in threat perception and marginally significant increase in worry)	Psychological	Encoding	No	-	**-**
	Exp 2 No	Neutral	Non-clinical	-	Psychological	Encoding	No	-	**-**
**Wang (2012)**	No	-	Non-clinical	-	N/A		N/A	-	**Response bias** & ***d′*** non-significant correlation between shyness with response bias and *d′*
**Curtis (2015)**	Exp 1 No	-	Non-clinical	Yes	Psychological	Encoding	Yes	-	***d′*** higher *d′* in control condition as compared to anxiety condition
	Exp 2 No	-	Non-clinical	Yes	Psychological	Retrieval	Yes	-	***d′*** no difference in *d′* between anxiety condition and control condition
**Attwood (2015)**	No	Neutral	Non-clinical	Yes	Physiological	Retrieval	Yes	-	**Hits** higher hits in control condition than anxiety condition **False-alarms, *d′* & response bias** no difference between anxiety condition and control condition for false-alarms, *d′* or response bias
**Kikutani (2018)**	Yes	Angry/Happy/Neutral	Non-clinical	Yes (higher social anxiety associated with smaller happiness advantage)	N/A		N/A	**General recognition accuracy** higher proportion correct for happy compared to angry; own-race faces: larger happiness advantage in participants with lower as compared to higher social anxiety; other-race faces: no difference in happiness advantage according to social anxiety	***d′*** no difference in *d′* according to social anxiety

*Note*. Not applicable: N/A, Not reported: -.

In the only study reporting on stimuli-valence [38], stimuli-valence interacted with the ORB, as individuals with higher as compared to lower social anxiety reported a reduced happiness advantage for happy own-race faces.

### Memory phase of anxiety induction

As seen in Table 3, use of a psychological induction (*n* = four) was more prevalent than physiological induction (*n* = two). The induction was successful in all studies examining the influence of anxiety induced during encoding, with the exception of experiment two in the study by Wilson et al. [67]. Further, the manipulation was also successful in the studies inducing anxiety during retrieval [19, 29, 30]. Only studies successfully inducing anxiety were reviewed due to the inability of determining anxiety impact without a successful manipulation of anxiety. In several studies, when anxiety was induced during encoding, recognition accuracy (*d′* or hits) was higher when experiencing lower anxiety as compared to higher anxiety [29, 63]. Further, false-alarms were lower when experiencing lower anxiety as compared to high in the study by Brigham et al [63].

For studies inducing anxiety during retrieval, only one study reported a difference in recognition accuracy between those experiencing high anxiety as compared to low anxiety [19]. This study [19] induced anxiety through physiological means. One study [30] that induced anxiety at retrieval did not report on the impact of this on face-recognition accuracy.

#### Use of STAI

Half of studies using an anxiety induction measured state and/or trait anxiety with the STAI (three out of six). Additionally, half of studies examining physiological anxiety used the STAI to measure induced anxiety (one out of two).

### Effect of race and interaction between anxiety and race

Studies examining the interaction between anxiety and race are presented in Table 4. All studies examining the relationship between anxiety and race recorded face-race and participant race and therefore controlled for the ORB. One study [30] found a significant negative correlation between anxiety and other-race stimuli according to general recognition accuracy. No elaboration on this interaction, however, was given. This study also found an increased hit-rate for other-race faces in the joy condition as compared to fear and control conditions. Most other studies examining recognition accuracy failed to find a significant interaction between anxiety and race [19, 29, 36, 38, 63].

**Table 4:** Race of participant and stimuli of studies and results of studies examining the interaction between anxiety and race.

First Author (year)		Face-stimuli race	Participant race	Results
**Brigham (1983)**		White (own-race)	White	***d′*, hits & false-alarms** no difference in *d′*, hits or false-alarms for own-race face-stimuli in high-anxiety condition as compared to other-race face-stimuli in moderate-anxiety condition; non-significant correlation between *d′* and racial attitudes and contact with other-race
		Black (other-race)		
**Johnson (2005)**	Exp 1	White (own-race)	White	**Exp 1 & 2**
				**General recognition accuracy** negative correlation between anxiety & other-race recognition accuracy
		Black (other-race)		
				**Hit-rate** higher hit-rate for other-race faces in joy condition as compared to fear and control conditions
	Exp 2	White (own-race)	White	
		Black (other-race)		**False-alarms** no interaction between anxiety and race or difference between false-alarms in joy, control, or fear conditions
				***d′*** reduced magnitude of ORB in joy condition as compared to fear condition and control conditions; higher recognition accuracy for other-race in joy condition as compared to fear and control conditions; no difference in own-race recognition accuracy between joy, control and fear condition; ORB found in control and fear condition, but no ORB found in joy condition; no difference in ORB between fear and control conditions
**Walker (2008)**		South Asian (own-race) White (own-race)	South Asian & White	***d′*** For White (own-race) participants: individuating experience significantly positively correlated with *d′* for South Asian (other-race) and marginally significant correlation with Black (other-race); For South Asian participants (own-race): individuating experience and contact significantly positively correlated with White (other-race) *d′* and individuating experience significantly positively correlated with *d′* for Black (other-race); For South Asian participants (own-race): intergroup anxiety significantly correlated with *d′* for White (other-race)
		Black (other-race)		
**Horry (2009)**	Exp 2	White (own-race)	White	**Hit-rate, false-alarm rate & *d′*** no interaction between anxiety and race
		Middle Eastern (other-race)		
**Wilson (2010)**	Exp 1	White (own-race)	White	**Exp 1 & 2**
		Hispanic (other-race)		***d′*** higher *d′* for own-race as compared to other-race in control condition; no difference in *d′* between other-race and own-race in intergroup threat condition; higher *d′* for own-race in control condition than in intergroup threat condition; no difference in *d′* for other-race between intergroup threat condition and control condition
	Exp 2	White (own-race)	White	
		Hispanic (other-race)		
**Wang (2012)**		East Asian (own-race)	East Asian	**Response bias** significant positive relationship between shyness and response criterion for own-race faces, with stricter response criterion for own-race faces in highly shy participants
		Black (other-race)		***d′*** no interaction between anxiety and race
		South Asian (other-race)		
**Curtis (2015)**	Exp 1	White (own-race)	White	**Response bias** more liberal criterion for other-race faces in control as compared to anxiety condition
		Black (other-race)		***d′*** no interaction between anxiety and race
	Exp 2	White (own-race)	White	**Response bias & *d′*** no interaction between anxiety and race
		Black (other-race)		
**Attwood (2015)**		White (own-race)	White	**Hits** & **false-alarms** no interaction between anxiety and race
		South Asian (other-race)		
**Kikutani (2018)**		Asian (own-race)	East Asian	**All recognition measures** no interaction between anxiety and race
		White (other-race)		

Some studies [29, 36], however, did find differences between face-recognition accuracy according to level of anxiety. In Horry et al. [36], a negative correlation occurred between anxiety and *d′* in the control condition, as more anxious participants were less accurate, with the opposite discovered in the anxiety condition. In Curtis et al. [29], however, when anxiety was induced during encoding in the anxiety condition, *d′* was significantly worse than the control condition.

## Discussion

The goal of this systematic literature review was to examine whether anxiety and race, when acting together, could affect face-recognition accuracy. Specifically, this review aimed to investigate whether results supported an interaction between anxiety and race, as predicted by Brigham [49]; or no interaction, but performance reductions, as suggested by ACT [8]. We also explored the influence of moderators affecting anxiety and race which could potentially influence this relationship. The potential influence of anxiety induced during encoding or retrieval was also examined as a way of elucidating whether anxiety can influence face-recognition accuracy when induced during both phases of memory.

### Relationship between anxiety and race

According to Brigham [49], anxiety and race can interact to influence face-recognition accuracy. Several studies reviewed, indicated support for this perspective [30, 47]. In the study by Johnson et al. [30] a negative correlation existed between anxiety and other-race recognition accuracy suggesting that anxiety reduced face-recognition accuracy for other-race faces. This result occurred despite no difference in the ORB arising between the fear condition and control condition. It must be noted, however, that the non-significant difference in the ORB between these conditions does not comment on the impact of anxiety. Fear and anxiety can be considered distinct affective states [73, 74]. Further, the video priming fear used in the fear condition might have omitted important components of anxiety in the response elicited, thereby impacting findings within this condition. Specifically, the video induction might not have provoked worry over future misfortune, which is a cognitive component of anxiety that creates rumination and the diversion of processing resources to worry thoughts [8]. Therefore, future studies examining the interaction between anxiety and race should use an induction specifically seeking to induce anxiety. Additionally, the correlational result between anxiety and race should be further examined as correlations do not allow for concrete conclusions regarding causality.

In Walker et al. [47], intergroup anxiety was also correlated with other-race (White) face-recognition accuracy in South Asian participants. In their discussion they indicated that intergroup anxiety impaired the ability to differentiate other-race faces through decreased attentional focus. It must be noted that the conclusion drawn from the results presented in Walker et al. [47] and that reported in the results section of their paper were inconsistent. The directionality of the correlation reported within Walker et al. [47] between intergroup anxiety and *d′* for other-race (White) individuals was indicated as positive, while their discussion suggested this was negative. Therefore, although their discussion does indicate support for the interaction proposed by Brigham [49], due to the discrepancy, the findings of Walker et al. [47] should be interpreted with caution.

The findings of Johnson et al. [30] specific to anxiety, and Walker et al. [47], as per their discussion, supports the theory adapted from Brigham [49]. The correlation between anxiety and race in Johnson et al. [30], separately from their findings regarding fear, also suggests that anxiety unrelated to the intergroup context can interact with race [29]. As said above, however, due to the correlational result of Johnson et al. [30] and the inconsistent findings of Walker et al [47], further research needs to be conducted to fully provide support for Brigham [49].

Proposing an alternative interaction to Brigham [49], Wilson et al. [67] found reduced own-race face-recognition accuracy in the threat condition as compared to control condition. The reduction in own-race recognition accuracy was so large in this study that recognition accuracy was comparable to other-race face-recognition accuracy. This finding was explained according to increased distinctiveness threat for own-race participants when feeling threatened by an outgroup encroaching on their ingroup distinctiveness [66]. This finding was not expected, nor predicted. Studies included in this review suggest that anxiety, under certain circumstances, can either increase [30, 47] or decrease [67] own-race or other-race face-recognition accuracy. Potentially, situations where ingroup distinctiveness is challenged could lead anxiety to impair own-race recognition accuracy. While anxiety provoking situations where ingroup distinctiveness is not challenged, might instead impair other-race face-recognition accuracy. Due to limited studies supporting either perspective and caution regarding the interpretation of findings, future research needs to explore this possibility using an experimental design capable of testing both theories.

Despite above findings indicating a potential interaction between anxiety and race, several other studies reviewed did not support an interaction. Curtis et al. [29] and Attwood et al. [19] reported a reduction in face-recognition accuracy when state anxious during encoding and retrieval, respectively. Neither study, however, found that face-recognition accuracy for other-race faces was influenced by state anxiety. These findings, therefore, supported ACT [8], as anxiety reduced face-recognition accuracy, but did not interact with race. Further, in several other studies reviewed [36, 38, 63], an interaction between anxiety and race also did not occur. Potential reasons for why these studies were unable to find an interaction between anxiety and race, are elaborated below. This discussion is centred around the impact of several moderators identified during the first two aims of this systematic literature review, which might have reduced the chance of finding an interaction. The impact of anxiety induced at either encoding or retrieval, on face-recognition accuracy is also explored at this point.

### Moderators

#### Face-stimuli valence

In our review, most studies either did not report on the impact of face-stimuli valence on face-recognition accuracy or used neutral face-stimuli. The only study examining the impact of face-stimuli valence [38] found that more socially anxious individuals had a less pronounced happiness advantage for own-race faces than those that were less anxious. Therefore, despite social anxiety impairing recognition for happy own-race faces when anxious, recognition accuracy for these faces was still higher than for angry faces. Although this finding is in contrast with previous research indicating preferential processing of negative stimuli [26, 27], it highlights that stimuli-valence can moderate the influence of anxiety on face-recognition accuracy [38]. Therefore, the lack of interaction between anxiety and race found in the study by Kikutani [38], might have occurred due to stimuli-valence impacting this relationship [75]. The uncontrolled stimuli-valence in several other studies reviewed [29, 36, 63, 68], might have also impaired the ability to find an interaction through masking effects caused by heightened error of measurement and statistical noise [75]. Future studies need to experimentally control for stimuli-valence when seeking to explore the interaction between anxiety and race.

#### Gender

Although most studies commenting on the influence of gender, did not find an impact of gender on face-recognition accuracy, these discussions were very brief and only mentioned without elaboration. In two studies reviewed, however, gender moderated the impact of anxiety and race on face-recognition accuracy. In the study by Brigham et al. [63], gender moderated the influence of anxiety on face-recognition accuracy through increased recognition accuracy for female participants within the moderate-anxiety condition compared to high-anxiety condition. Further, gender also moderated the own-race bias in Johnson et al. [30] with higher recognition accuracy for female own-race face-stimuli compared to all other types of face-stimuli. Additionally, although not reflected strongly by the results of this review, extensive evidence indicates the presence of an own-gender bias within female participants [45]. Therefore, like with stimuli-valence, gender might also have moderated the relationship between anxiety and race through masking effects [75]. Indeed, within several studies [19, 68] reviewed where an interaction between anxiety and race did not occur, the impact of gender was not examined. Therefore, future studies need to control for gender by either using same gender participants and stimuli, or by purposely controlling for gender experimentally.

#### Contact and individuating experience

In support of the review article by Brigham [49], perceptual expertise (specifically contact and individuating experience) also interacted with face-recognition accuracy for different race faces [47] such that increased contact was related to better face-recognition accuracy. Despite this, an interaction between anxiety and race in Walker et al. [47] was still present. This suggests that increased perceptual expertise could have facilitated an interaction between anxiety and race, through strengthening the impact of race. Therefore, future research should measure the moderating influence of perceptual expertise with other-race individuals in future studies.

#### Characteristics of included studies

Other factors associated with characteristics of included studies might have also impacted the potential of finding an interaction between anxiety and race. Although most studies used a racial face-recognition paradigm including both an encoding; learning phase, and retrieval; test phase, several others did not [36, 47]. In Horry et al. [36], faces were initially presented through a dot-probe task [76]. Although this design is effective for looking at attentional biases, it is a suboptimal method for examining face-recognition accuracy, as faces are presented concurrently [29].

As seen above, all studies which did not find an interaction [19, 29, 36, 38, 63] between anxiety and race, were potentially impacted by at least one moderator indicated within this review. Specifically, face-stimuli valence [29, 36, 38, 63], gender [19], or methodological characteristics [36]. Therefore, until these moderators are accounted for, it is unclear whether an interaction between anxiety and race can occur.

#### Memory phase of anxiety induction

Separately from the interaction between anxiety and race and relevant moderators, this review examined the influence of anxiety on encoding and retrieval. In several reviewed studies, anxiety induced during encoding influenced face-recognition accuracy. These results corroborate with Deffenbacher et al. [5], who stated that anxiety may only influence encoding. In reviewed studies examining the influence of anxiety induced during encoding, most studies found an increased *d′* or hits when experiencing lower anxiety [29, 63], with increased false alarms when experiencing heightened anxiety [63]. These results also support ACT, as participants experiencing lower levels of anxiety had increased cognitive performance compared to those subjected to increased anxiety [8].

Contrary to Deffenbacher et al. [7] who suggested that anxiety may only influence face-recognition accuracy during encoding, the study by Attwood et al. [19] discovered that anxiety induced during retrieval could also influence face-recognition accuracy. Why anxiety induced during retrieval reduced face-recognition accuracy is unclear. Speculation suggests that these findings occurred because of fundamental differences existing between the anxiety manipulations used in these studies. In Attwood et al. [19], anxiety was altered through physiological means, as state anxiety was induced through the 7.5% CO_2_ challenge. In Curtis et al. [29], anxiety did not affect face-recognition accuracy; however, anxiety was induced during retrieval through a cognitive anxiety induction.

Cognitive manipulations rely on a learned anxiety reaction [77]. That is, to become state anxious to a cognitive manipulation, such as a public speaking task (e.g. [29]), individuals need to have learned through experience to become state anxious to this stressor. Physiological anxiety manipulations, on the other hand, elicit an unconditioned or innate response [17, 18, 77], leading to a stronger overall reaction. Therefore, the fact that a physiological stressor reduced face-recognition accuracy during retrieval, and a cognitive stressor did not, suggests that a larger anxiety response is needed to disturb the retrieval, as compared to encoding phase of memory. This conclusion is supported by the finding that memory retrieval is strongly influenced by acute anxiety [78].

Results of the current review, therefore, suggest that anxiety can influence face-recognition accuracy during retrieval when the anxiety manipulation is physiological. Future research should test this hypothesis by comparing the anxiety response elicited by psychological and physiological anxiety inductions during both encoding and retrieval. Such research should be done while also measuring if individuals reacting less to psychological inductions and having lower anxiety, produce smaller indicators of anxiety (both physiological and psychological). This measurement should be done when using an anxiety scale specifically measuring physiological anxiety, such as the State-Trait Inventory for Cognitive and Somatic Anxiety (STICSA) [11]. Half of studies in this review measured physiological anxiety using the STAI [13] which has been criticised for not capturing anxiety related to physiological symptoms [11, 79].

### Limitations

Several limitations were associated with how the current review was conducted and existed within the studies themselves. Although most studies reviewed used signal detection measures, some did not. Heterogeneity also existed between studies in terms of the type of anxiety examined (i.e., social anxiety or intergroup anxiety). Further, in some studies reviewed, the manipulations used to induce anxiety also induced other affective states, such as fear. Additionally, not all studies included used a face-recognition paradigm for both the encoding and retrieval phases which might have also influenced results.

## Conclusions

Anxiety was found to reduce face-recognition accuracy when induced during either encoding or retrieval. Physiological anxiety was suggested to have a better chance of disrupting retrieval due to not relying on a learned anxiety response [77]. In our review, anxiety was found to disrupt cognitive performance and subsequently influence face-recognition accuracy, therefore supporting ACT [8]. Although some results supported Brigham [49] related to race-specific recognition errors elicited by anxiety, support for this perspective was limited. Further, in one study reviewed [67], an interaction occurred between anxiety and race; however, the direction was contrary to that expected by Brigham [49]. Several moderators were identified that potentially affected the relationship between anxiety, race, and face-recognition accuracy. These moderators could potentially have reduced or altered the ability to find an interaction between these factors. Until potential moderators are addressed by future research, it cannot be adequately concluded whether anxiety and race can combine to affect face-recognition accuracy, and the direction of this relationship.

## Supporting information

S1 Table PRISMA checklist. (DOC)

S2 Table Risk of bias summary for included studies. (DOCX)

S1 File Footnote 1. (DOCX)

S1 Appendix Search strategies. (DOCX)
