# Supplementary material for: Can anxiety and race interact to influence face-recognition accuracy? A systematic literature review
Source: PLoS One. 2021 Aug 6;16(8):e0254477. doi: 10.1371/journal.pone.0254477 (PMC8345850; doi:10.1371/journal.pone.0254477)
Supplement: S2 Table — (DOCX) [file pone.0254477.s002.docx]

**S2 Table. Risk of Bias Summary for included studies.**

|  | **Random Sequence generation (selection bias)** | **Allocation concealment (selection bias)** | **Blinding of participants (performance bias)** | **Blinding of outcome assessment (detection bias)** | **Incomplete outcome data addressed (attrition bias)** | **Selective reporting (reporting bias)** |
| --- | --- | --- | --- | --- | --- | --- |
| **Anxiety x Race Interaction** |  |  |  |  |  |  |
| Brigham et al. (1983) | **+** | **+** | **+** | **+** | **+** | **+** |
| Johnson et al. (2005) | **+** | **?** | **+** | **+** | **-** | **+** |
| Walker et al. (2008) | **?** | **?** | **?** | **+** | **+** | **+** |
| Horry et al. (2009) | **+** | **?** | **+** | **+** | **+** | **-** |
| Wilson et al. (2010) | **+** | **?** | **+** | **+** | **?** | **+** |
| Wang et al. (2012) | **?** | **?** | **+** | **+** | **+** | **?** |
| Curtis et al. (2015) | **+** | **?** | **+** | **+** | **+** | **+** |
| Attwood et al. (2015) | **+** | **+** | **+** | **+** | **+** | **-** |
| Kikutani (2018) | **+** | **?** | **+** | **+** | **+** | **?** |
