# Supplementary material for: Can anxiety and race interact to influence face-recognition accuracy? A systematic literature review
Source: PLoS One. 2021 Aug 6;16(8):e0254477. doi: 10.1371/journal.pone.0254477 (PMC8345850; doi:10.1371/journal.pone.0254477)
Supplement: S1 File — (DOCX) [file pone.0254477.s003.docx]

**Footnote 1.** Although searching with Science Direct was also indicated via the pre-registered protocol, due to the complexity of the current search and overlap with Scopus, upon recommendation from the librarian this database was excluded.
