# Supplementary material for: Can anxiety and race interact to influence face-recognition accuracy? A systematic literature review
Source: PLoS One. 2021 Aug 6;16(8):e0254477. doi: 10.1371/journal.pone.0254477 (PMC8345850; doi:10.1371/journal.pone.0254477)
Supplement: S1 Appendix — (DOCX) [file pone.0254477.s004.docx]

**S1 Appendix.** Search Strategies

**Web of Science: 11359 (7/02/2020)**

((TS=(anxi* OR angst OR "cognitive* anxi*" OR "psychological* anxi*" OR "somatic* anxi*" OR "physical* anxi*" OR "physiological* anxi*" OR "physical* stress*" OR "physiological* stress*" OR "mental* stress*" OR "psychological* stress*" OR stress* OR worry OR worrie* OR nervous* OR apprehens* OR "psychological* arous*" OR "physiological* arous*" OR "physical* arous*" OR "mental* arous*" OR arous* OR distress* OR "affect* arousal" OR affective OR affect OR "7.5% carbon dioxide" OR "7.5% CO2" OR neuroticism)) AND (TS=("fac* perception" OR "perception of fac*" OR "fac* recognition" OR "recognition of fac*" OR "fac* memory" OR "memory for fac*" OR "fac* identity" OR "identity of fac*" OR "fac* identification" OR "identification of fac*" OR "fac* processing" OR "processing of fac*" OR "recognition accuracy" OR "recognition memory" OR "eyewitness accuracy" OR "eyewitness memory" OR "eyewitness identification" OR "eyewitness id" OR "identity verification" OR "identification verification" OR "id verification" OR "identity parade" OR "identification decision accuracy" OR "identification accuracy" OR "witness accuracy" OR "witness memory" OR "witness identification" OR "witness id" OR "witness verification" OR "familiarity judgement*" OR "familiarity recognition" OR lineup OR "line up" OR "photo array" OR "photo display" OR "photo spread" OR photospread))) AND LANGUAGE: (English)

Timespan: All years. Indexes: SCI-EXPANDED, SSCI, A&HCI, CPCI-S, CPCI-SSH, BKCI-S, BKCI-SSH, ESCI, CCR-EXPANDED, IC.

**PsycArticles (Ovid): 659 (7/02/2020)**

| Search number | Searches | Results |
| --- | --- | --- |
| 1 | ((anxi* or angst or cognitive* anxi* or psychological* anxi* or somatic* anxi* or physical* anxi* or physiological* anxi* or physical* stress* or physiological* stress* or mental* stress* or psychological* stress* or stress* or worry or worrie* or nervous* or apprehens* or psychological* arous* or physiological* arous* or physical* arous* or mental* arous* or arous* or distress* or affect* arousal or affect or affective or 7$3 carbon dioxide or 7$3 ?CO2? or neuroticism) and ((fac* adj2 perception) or (fac* adj2 recognition) or (fac* adj2 memory) or (fac* adj2 identity) or (fac* adj2 identification) or (fac* adj2 processing) or recognition accuracy or recognition memory or eyewitness accuracy or eyewitness memory or eyewitness identification or eyewitness id or identity verification or identification verification or id verification or identity parade or identification decision accuracy or identification accuracy or witness accuracy or witness memory or witness identification or witness id or witness verification or familiarity judgement* or familiarity recognition or lineup or line up or photo array or photo display or photo spread or photospread)).ab. | 640 |
| 2 | \| limit 1 to psycarticles \|  \| \| --- \| --- \| | 614 |
| 3 | ((anxi* or angst or cognitive* anxi* or psychological* anxi* or somatic* anxi* or physical* anxi* or physiological* anxi* or physical* stress* or physiological* stress* or mental* stress* or psychological* stress* or stress* or worry or worrie* or nervous* or apprehens* or psychological* arous* or physiological* arous* or physical* arous* or mental* arous* or arous* or distress* or affect* arousal or affect or affective or 7$3 carbon dioxide or 7$3 ?CO2? or neuroticism) and ((fac* adj2 perception) or (fac* adj2 recognition) or (fac* adj2 memory) or (fac* adj2 identity) or (fac* adj2 identification) or (fac* adj2 processing) or recognition accuracy or recognition memory or eyewitness accuracy or eyewitness memory or eyewitness identification or eyewitness id or identity verification or identification verification or id verification or identity parade or identification decision accuracy or identification accuracy or witness accuracy or witness memory or witness identification or witness id or witness verification or familiarity judgement* or familiarity recognition or lineup or line up or photo array or photo display or photo spread or photospread)).ti. | 54 |
| 4 | limit 1 to psycarticles | 49 |
| 5 | ((anxi* or angst or cognitive* anxi* or psychological* anxi* or somatic* anxi* or physical* anxi* or physiological* anxi* or physical* stress* or physiological* stress* or mental* stress* or psychological* stress* or stress* or worry or worrie* or nervous* or apprehens* or psychological* arous* or physiological* arous* or physical* arous* or mental* arous* or arous* or distress* or affect* arousal or affect or affective or 7$3 carbon dioxide or 7$3 ?CO2? or neuroticism) and ((fac* adj2 perception) or (fac* adj2 recognition) or (fac* adj2 memory) or (fac* adj2 identity) or (fac* adj2 identification) or (fac* adj2 processing) or recognition accuracy or recognition memory or eyewitness accuracy or eyewitness memory or eyewitness identification or eyewitness id or identity verification or identification verification or id verification or identity parade or identification decision accuracy or identification accuracy or witness accuracy or witness memory or witness identification or witness id or witness verification or familiarity judgement* or familiarity recognition or lineup or line up or photo array or photo display or photo spread or photospread)).kw. | 26 |
| 6 | limit 1 to psycarticles | 25 |
| 9 | 2 or 4 or 6 | 659 |

**PsycINFO (Ovid): 4232 (7/02/2020)**

| Search number | Searches | Results |
| --- | --- | --- |
| 1 | ((anxi* or angst or cognitive* anxi* or psychological* anxi* or somatic* anxi* or physical* anxi* or physiological* anxi* or physical* stress* or physiological* stress* or mental* stress* or psychological* stress* or stress* or worry or worrie* or nervous* or apprehens* or psychological* arous* or physiological* arous* or physical* arous* or mental* arous* or arous* or distress* or affect* arousal or affect or affective or 7$3 carbon dioxide or 7$3 ?CO2? or neuroticism) and ((fac* adj2 perception) or (fac* adj2 recognition) or (fac* adj2 memory) or (fac* adj2 identity) or (fac* adj2 identification) or (fac* adj2 processing) or recognition accuracy or recognition memory or eyewitness accuracy or eyewitness memory or eyewitness identification or eyewitness id or identity verification or identification verification or id verification or identity parade or identification decision accuracy or identification accuracy or witness accuracy or witness memory or witness identification or witness id or witness verification or familiarity judgement* or familiarity recognition or lineup or line up or photo array or photo display or photo spread or photospread)).ab. | 5121 |
| 2 | limit 1 to (peer reviewed journal and English language) | 3937 |
| 3 | ((anxi* or angst or cognitive* anxi* or psychological* anxi* or somatic* anxi* or physical* anxi* or physiological* anxi* or physical* stress* or physiological* stress* or mental* stress* or psychological* stress* or stress* or worry or worrie* or nervous* or apprehens* or psychological* arous* or physiological* arous* or physical* arous* or mental* arous* or arous* or distress* or affect* arousal or affect or affective or 7$3 carbon dioxide or 7$3 ?CO2? or neuroticism) and ((fac* adj2 perception) or (fac* adj2 recognition) or (fac* adj2 memory) or (fac* adj2 identity) or (fac* adj2 identification) or (fac* adj2 processing) or recognition accuracy or recognition memory or eyewitness accuracy or eyewitness memory or eyewitness identification or eyewitness id or identity verification or identification verification or id verification or identity parade or identification decision accuracy or identification accuracy or witness accuracy or witness memory or witness identification or witness id or witness verification or familiarity judgement* or familiarity recognition or lineup or line up or photo array or photo display or photo spread or photospread)).ti. | 551 |
| 4 | limit 3 to (peer reviewed journal and English language) | 460 |
| 5 | ((anxi* or angst or cognitive* anxi* or psychological* anxi* or somatic* anxi* or physical* anxi* or physiological* anxi* or physical* stress* or physiological* stress* or mental* stress* or psychological* stress* or stress* or worry or worrie* or nervous* or apprehens* or psychological* arous* or physiological* arous* or physical* arous* or mental* arous* or arous* or distress* or affect* arousal or affect or affective or 7$3 carbon dioxide or 7$3 ?CO2? or neuroticism) and ((fac* adj2 perception) or (fac* adj2 recognition) or (fac* adj2 memory) or (fac* adj2 identity) or (fac* adj2 identification) or (fac* adj2 processing) or recognition accuracy or recognition memory or eyewitness accuracy or eyewitness memory or eyewitness identification or eyewitness id or identity verification or identification verification or id verification or identity parade or identification decision accuracy or identification accuracy or witness accuracy or witness memory or witness identification or witness id or witness verification or familiarity judgement* or familiarity recognition or lineup or line up or photo array or photo display or photo spread or photospread)).id | 676 |
| 6 | limit 5 to (peer reviewed journal and English language) | 534 |
| 7 | ((anxi* or angst or cognitive* anxi* or psychological* anxi* or somatic* anxi* or physical* anxi* or physiological* anxi* or physical* stress* or physiological* stress* or mental* stress* or psychological* stress* or stress* or worry or worrie* or nervous* or apprehens* or psychological* arous* or physiological* arous* or physical* arous* or mental* arous* or arous* or distress* or affect* arousal or affect or affective or 7$3 carbon dioxide or 7$3 ?CO2? or neuroticism) and ((fac* adj2 perception) or (fac* adj2 recognition) or (fac* adj2 memory) or (fac* adj2 identity) or (fac* adj2 identification) or (fac* adj2 processing) or recognition accuracy or recognition memory or eyewitness accuracy or eyewitness memory or eyewitness identification or eyewitness id or identity verification or identification verification or id verification or identity parade or identification decision accuracy or identification accuracy or witness accuracy or witness memory or witness identification or witness id or witness verification or familiarity judgement* or familiarity recognition or lineup or line up or photo array or photo display or photo spread or photospread)).sh. | 34 |
| 8 | limit 3 to (peer reviewed journal and English language) | 28 |
| 9 | 2 or 4 or 6 or 8 | **4232** |

**ProQuest: 2713 (7/02/2020)**

Limit results to English language, full text and peer reviewed.

**Title and Abstract:** 2532

AB,TI(anxi* OR angst OR "cognitive* anxi*" OR "psychological* anxi*" OR "somatic* anxi*" OR "physical* anxi*" OR "physiological* anxi*" OR "physical* stress*" OR "physiological* stress*" OR "mental* stress*" OR "psychological* stress*" OR stress* OR worry OR worrie* OR nervous* OR apprehens* OR "psychological* arous*" OR "physiological* arous*" OR "physical* arous*" OR "mental* arous*" OR arous* OR distress* OR "affect* arousal" OR affective OR affect OR "7.5% carbon dioxide" OR "7.5% CO2" OR neuroticism) AND AB,TI(fac* NEAR/2 perception OR fac* NEAR/2 recognition OR fac* NEAR/2 memory OR fac* NEAR/2 identity OR fac* NEAR/2 identification OR fac* NEAR/2 processing OR "recognition accuracy" OR "recognition memory" OR "eyewitness accuracy" OR "eyewitness memory" OR "eyewitness identification" OR "eyewitness id" OR "identity verification" OR "identification verification" OR "id verification" OR "identity parade" OR "identification decision accuracy" OR "identification accuracy" OR "witness accuracy" OR "witness memory" OR "witness identification" OR "witness id" OR "witness verification" OR "familiarity judgement*" OR "familiarity recognition" OR lineup OR "line up" OR "photo array" OR "photo display" OR "photo spread" OR photospread)

**Keywords and Subject headings:** 181

IF,SU(anxi* OR angst OR "cognitive* anxi*" OR "psychological* anxi*" OR "somatic* anxi*" OR "physical* anxi*" OR "physiological* anxi*" OR "physical* stress*" OR "physiological* stress*" OR "mental* stress*" OR "psychological* stress*" OR stress* OR worry OR worrie* OR nervous* OR apprehens* OR "psychological* arous*" OR "physiological* arous*" OR "physical* arous*" OR "mental* arous*" OR arous* OR distress* OR "affect* arousal" OR affective OR affect OR "7.5% carbon dioxide" OR "7.5% CO2" OR neuroticism) AND IF,SU(fac* NEAR/2 perception OR fac* NEAR/2 recognition OR fac* NEAR/2 memory OR fac* NEAR/2 identity OR fac* NEAR/2 identification OR fac* NEAR/2 processing OR "recognition accuracy" OR "recognition memory" OR "eyewitness accuracy" OR "eyewitness memory" OR "eyewitness identification" OR "eyewitness id" OR "identity verification" OR "identification verification" OR "id verification" OR "identity parade" OR "identification decision accuracy" OR "identification accuracy" OR "witness accuracy" OR "witness memory" OR "witness identification" OR "witness id" OR "witness verification" OR "familiarity judgement*" OR "familiarity recognition" OR lineup OR "line up" OR "photo array" OR "photo display" OR "photo spread" OR photospread)

**Scopus: 7218 (7/02/2020)**

( TITLE-ABS-KEY ( anxi*  OR  angst  OR  "cognitive* anxi*"  OR  "psychological* anxi*"  OR  "somatic* anxi*"  OR  "physical* anxi*"  OR  "physiological* anxi*"  OR  "physical* stress*"  OR  "physiological* stress*"  OR  "mental* stress*"  OR  "psychological* stress*"  OR  stress* OR  worry  OR  worrie*  OR  nervous*  OR  apprehens*  OR  "psychological* arous*"  OR  "physiological* arous*"  OR  "physical* arous*"  OR  "mental* arous*"  OR  arous*  OR  distress*  OR  "affect* arousal"  OR  affective  OR  affect  OR  "7.5% carbon dioxide"  OR  "7.5% CO2"  OR  neuroticism ) )  AND  ( TITLE-ABS-KEY ( fac*  W/2  perception  OR  fac*  W/2  recognition  OR  fac*  W/2  memory  OR  fac*  W/2  identity  OR  fac*  W/2  identification  OR  fac*  W/2  processing  OR  "recognition accuracy"  OR  "recognition memory"  OR  "eyewitness accuracy"  OR  "eyewitness memory"  OR  "eyewitness identification"  OR  "eyewitness id"  OR  "identity verification"  OR  "identification verification"  OR  "id verification"  OR  "identity parade"  OR  "identification decision accuracy"  OR  "identification accuracy"  OR  "witness accuracy"  OR  "witness memory"  OR  "witness identification"  OR  "witness id"  OR  "witness verification"  OR  "familiarity judgement*"  OR  "familiarity recognition"  OR  lineup  OR  "line up"  OR  "photo array"  OR  "photo display"  OR  "photo spread"  OR  photospread) )  AND  ( LIMIT-TO ( LANGUAGE ,  "English" ) )

**Wiley Online Library: 1366 (7/02/2020)**

**Abstract: 1281**

"anxi* OR angst OR "cognitive anxiety" OR "cognitively anxious" OR "psychological anxiety" OR "psychologically anxious" OR "somatic anxiety" OR "somatically anxious" OR "physical anxiety" OR "physically anxious" OR "physiological anxiety" OR "physiologically anxious" OR "physical stress" OR "physically stressed" OR "physically stressing" OR "physiological stress" OR "physiologically stressed" OR "physiologically stressing" OR "mental stress" OR "mentally stressed" OR "mentally stressing" OR "psychological stress" OR "psychologically stressed" OR "psychologically stressing" OR stress* OR worrie* OR worry OR nervous* OR apprehens* OR "psychological arousal" OR "psychologically aroused" OR "psychologically arousing" OR "physiological arousal" OR "physiologically aroused" OR "physiologically arousing" OR "physical arousal" OR "physically aroused" OR "physically arousing" OR "mental arousal" OR "mentally aroused" OR "mentally arousing" OR arous* OR distress* OR "affect arousal" OR "affective arousal" OR affective OR affect OR "7.5% carbon dioxide" OR "7.5% CO2" OR neuroticism" in Abstract and ""face perception" OR "facial perception" OR "perception of face" OR "perception of facial" OR "face recognition" OR "facial recognition" OR "recognition of face" OR "recognition of facial" OR "face memory" OR "facial memory" OR "memory for face" OR "memory for facial" OR "face identity" OR "facial identity" OR "identity of face" OR "identity of facial" OR "face identification" OR "facial identification" OR "identification of face" OR "identification of facial" OR "face processing" OR "facial processing" OR "processing of face" OR "processing of facial" OR "recognition accuracy" OR "recognition memory" OR "eyewitness accuracy" OR "eyewitness memory" OR "eyewitness identification" OR "eyewitness id" OR "identity verification" OR "identification verification" OR "id verification" OR "identity parade" OR "identification decision accuracy" OR "identification accuracy" OR "witness accuracy" OR "witness memory" OR "witness identification" OR "witness id" OR "witness verification" OR "familiarity judgement" OR "familiarity judgements" OR "familiarity recognition" OR lineup OR "line up" OR "photo array" OR "photo display" OR "photo spread" OR photospread" in Abstract

**Title: 65**

"anxi* OR angst OR "cognitive anxiety" OR "cognitively anxious" OR "psychological anxiety" OR "psychologically anxious" OR "somatic anxiety" OR "somatically anxious" OR "physical anxiety" OR "physically anxious" OR "physiological anxiety" OR "physiologically anxious" OR "physical stress" OR "physically stressed" OR "physically stressing" OR "physiological stress" OR "physiologically stressed" OR "physiologically stressing" OR "mental stress" OR "mentally stressed" OR "mentally stressing" OR "psychological stress" OR "psychologically stressed" OR "psychologically stressing" OR stress* OR worrie* OR worry OR nervous* OR apprehens* OR "psychological arousal" OR "psychologically aroused" OR "psychologically arousing" OR "physiological arousal" OR "physiologically aroused" OR "physiologically arousing" OR "physical arousal" OR "physically aroused" OR "physically arousing" OR "mental arousal" OR "mentally aroused" OR "mentally arousing" OR arous* OR distress* OR "affect arousal" OR "affective arousal" OR affective OR affect OR "7.5% carbon dioxide" OR "7.5% CO2" OR neuroticism" in Title and ""face perception" OR "facial perception" OR "perception of face" OR "perception of facial" OR "face recognition" OR "facial recognition" OR "recognition of face" OR "recognition of facial" OR "face memory" OR "facial memory" OR "memory for face" OR "memory for facial" OR "face identity" OR "facial identity" OR "identity of face" OR "identity of facial" OR "face identification" OR "facial identification" OR "identification of face" OR "identification of facial" OR "face processing" OR "facial processing" OR "processing of face" OR "processing of facial" OR "recognition accuracy" OR "recognition memory" OR "eyewitness accuracy" OR "eyewitness memory" OR "eyewitness identification" OR "eyewitness id" OR "identity verification" OR "identification verification" OR "id verification" OR "identity parade" OR "identification decision accuracy" OR "identification accuracy" OR "witness accuracy" OR "witness memory" OR "witness identification" OR "witness id" OR "witness verification" OR "familiarity judgement" OR "familiarity judgements" OR "familiarity recognition" OR lineup OR "line up" OR "photo array" OR "photo display" OR "photo spread" OR photospread" in Title

**Keywords: 20**

"anxi* OR angst OR "cognitive anxiety" OR "cognitively anxious" OR "psychological anxiety" OR "psychologically anxious" OR "somatic anxiety" OR "somatically anxious" OR "physical anxiety" OR "physically anxious" OR "physiological anxiety" OR "physiologically anxious" OR "physical stress" OR "physically stressed" OR "physically stressing" OR "physiological stress" OR "physiologically stressed" OR "physiologically stressing" OR "mental stress" OR "mentally stressed" OR "mentally stressing" OR "psychological stress" OR "psychologically stressed" OR "psychologically stressing" OR stress* OR worrie* OR worry OR nervous* OR apprehens* OR "psychological arousal" OR "psychologically aroused" OR "psychologically arousing" OR "physiological arousal" OR "physiologically aroused" OR "physiologically arousing" OR "physical arousal" OR "physically aroused" OR "physically arousing" OR "mental arousal" OR "mentally aroused" OR "mentally arousing" OR arous* OR distress* OR "affect arousal" OR "affective arousal" OR affective OR affect OR "7.5% carbon dioxide" OR "7.5% CO2" OR neuroticism" in Keywords and ""face perception" OR "facial perception" OR "perception of face" OR "perception of facial" OR "face recognition" OR "facial recognition" OR "recognition of face" OR "recognition of facial" OR "face memory" OR "facial memory" OR "memory for face" OR "memory for facial" OR "face identity" OR "facial identity" OR "identity of face" OR "identity of facial" OR "face identification" OR "facial identification" OR "identification of face" OR "identification of facial" OR "face processing" OR "facial processing" OR "processing of face" OR "processing of facial" OR "recognition accuracy" OR "recognition memory" OR "eyewitness accuracy" OR "eyewitness memory" OR "eyewitness identification" OR "eyewitness id" OR "identity verification" OR "identification verification" OR "id verification" OR "identity parade" OR "identification decision accuracy" OR "identification accuracy" OR "witness accuracy" OR "witness memory" OR "witness identification" OR "witness id" OR "witness verification" OR "familiarity judgement" OR "familiarity judgements" OR "familiarity recognition" OR lineup OR "line up" OR "photo array" OR "photo display" OR "photo spread" OR photospread" in Keywords

**PubMed: 2711 (7/02/2020)**

**Full text and English filter on.**

(anxi*[Title/Abstract] OR angst[Title/Abstract] OR "cognitive anxiety"[Title/Abstract] OR "cognitively anxious"[Title/Abstract] OR "psychological anxiety"[Title/Abstract] OR "psychologically anxious"[Title/Abstract] OR "somatic anxiety"[Title/Abstract] OR "somatically anxious"[Title/Abstract] OR "physical anxiety"[Title/Abstract] OR "physically anxious"[Title/Abstract] OR "physiological anxiety"[Title/Abstract] OR "physiologically anxious"[Title/Abstract] OR "physical stress"[Title/Abstract] OR "physically stress*"[Title/Abstract] OR "physiological stress"[Title/Abstract] OR "physiologically stress*"[Title/Abstract] OR "mental stress"[Title/Abstract] OR "mentally stress*"[Title/Abstract] OR "psychological stress"[Title/Abstract] OR "psychologically stress*"[Title/Abstract] OR stress*[Title/Abstract] OR worrie*[Title/Abstract] OR worry[Title/Abstract] OR nervous*[Title/Abstract] OR apprehens*[Title/Abstract] OR "psychological arousal"[Title/Abstract] OR "psychologically arous*"[Title/Abstract] OR "physiological arousal"[Title/Abstract] OR "physiologically arous*"[Title/Abstract] OR "physical arousal"[Title/Abstract] OR "physically arous*"[Title/Abstract] OR "mental arousal"[Title/Abstract] OR "mentally arous*"[Title/Abstract] OR arous*[Title/Abstract] OR distress*[Title/Abstract] OR "affect arousal"[Title/Abstract] OR "affective arousal"[Title/Abstract] OR affective[Title/Abstract] OR affect[Title/Abstract] OR "7.5% carbon dioxide"[Title/Abstract] OR "7.5% CO2"[Title/Abstract] OR neuroticism[Title/Abstract]) AND ("face perception"[Title/Abstract] OR "facial perception"[Title/Abstract] OR "perception of face"[Title/Abstract] OR "perception of facial"[Title/Abstract] OR "face recognition"[Title/Abstract] OR "facial recognition"[Title/Abstract] OR "recognition of face"[Title/Abstract] OR "recognition of facial"[Title/Abstract] OR "face memory"[Title/Abstract] OR "facial memory"[Title/Abstract] OR "memory for face"[Title/Abstract] OR "memory for facial"[Title/Abstract] OR "face identity"[Title/Abstract] OR "facial identity"[Title/Abstract] OR "identity of face"[Title/Abstract] OR "identity of facial"[Title/Abstract] OR "face identification"[Title/Abstract] OR "facial identification"[Title/Abstract] OR "identification of face"[Title/Abstract] OR "identification of facial"[Title/Abstract] OR "face processing"[Title/Abstract] OR "facial processing"[Title/Abstract] OR "processing of face"[Title/Abstract] OR "processing of facial"[Title/Abstract] OR "recognition accuracy"[Title/Abstract] OR "recognition memory"[Title/Abstract] OR "eyewitness accuracy"[Title/Abstract] OR "eyewitness memory"[Title/Abstract] OR "eyewitness identification"[Title/Abstract] OR "eyewitness id"[Title/Abstract] OR "identity verification"[Title/Abstract] OR "identification verification"[Title/Abstract] OR "id verification"[Title/Abstract] OR "identity parade"[Title/Abstract] OR "identification decision accuracy"[Title/Abstract] OR "identification accuracy"[Title/Abstract] OR "witness accuracy"[Title/Abstract] OR "witness memory"[Title/Abstract] OR "witness identification"[Title/Abstract] OR "witness id"[Title/Abstract] OR "witness verification"[Title/Abstract] OR "familiarity judgement*"[Title/Abstract] OR "familiarity recognition"[Title/Abstract] OR lineup[Title/Abstract] OR "line up"[Title/Abstract] OR "photo array"[Title/Abstract] OR "photo display"[Title/Abstract] OR "photo spread"[Title/Abstract] OR photospread[Title/Abstract])
